# Quantifying Stress Shielding in Dental Implants: A Comparative Finite Element Study of Titanium, CFR-PEEK, and Ceramic Materials

**DOI:** 10.3390/ma19050869

**Published:** 2026-02-26

**Authors:** Mario Ceddia, Tea Romasco, Natalia Di Pietro, Alessandro Cipollina, Adriano Piattelli, Luciano Lamberti, Bartolomeo Trentadue

**Affiliations:** 1 Department of Mechanics, Mathematics and Management, Polytechnic University of Bari, 70125 Bari, Italy; luciano.lamberti@poliba.it (L.L.); bartolomeo.trentadue@poliba.it (B.T.); 2 Department of Medical, Oral and Biotechnological Sciences, “G. D’Annunzio” University of Chieti-Pescara, 66100 Chieti, Italy; tea.romasco@unich.it (T.R.); natalia.dipietro@unich.it (N.D.P.); 3 Independent Researcher, 92019 Sciacca, Italy; alexandros1960@libero.it; 4 School of Dentistry, Saint Camillus International University of Health and Medical Sciences, 00131 Rome, Italy; apiattelli51@gmail.com; 5 Facultad de Medicina, UCAM Universidad Católica San Antonio de Murcia, 30107 Murcia, Spain

**Keywords:** stress shielding, dental implant biomechanics, FEA, PEEK polymer, Y-TZP, titanium

## Abstract

**Background**: Stress shielding, which occurs when there is a mismatch between the stiffness of the implant and the bone, can alter load transfer and drive peri-implant bone remodeling, particularly in low-density bone. **Methods**: We compared the biomechanical responses of one-piece implants made of Ti-6Al-4V, Y-TZP, and CFR-PEEK. We modelled the bone as linearly isotropic in the transverse direction and the implants as linearly isotropic with a fully bonded interface. A static load of 200 N was applied at an inclination of 30° buccal-to-lingual, with the transverse bone bottom faces fully constrained. **Results**: The peak cortical von Mises stress was highest for Y-TZP (87 MPa), followed by Ti-6Al-4V (57 MPa) and CFR-PEEK (approximately 37 MPa). Peak cortical von Mises strain showed the same relative order of magnitude: 3450 µε, 3103 µε, and 1523 µε, respectively. The stress-shielding factor (SSF) revealed that shielding was present in the mid-apical regions. Y-TZP exhibited the greatest shielding (SSF: 0.844–0.877), followed by Ti-6Al-4V (SSF: 0.380–0.568) and CFR-PEEK (SSF: 0.375–0.437). No crestal shielding was observed (SSF < 0). **Conclusions**: Overall, implants with higher stiffness increased crestal stress concentration and deepened peri-implant shielding. Meanwhile, CFR-PEEK improved load sharing and produced a more homogeneous mechanical stimulus in low-density bone.

## 1. Introduction

Modern implant dentistry is associated with high, predictable clinical outcomes. In a systematic review, Van den Breemer et al. [1] reported that the five-year survival rates for glass ceramics and feldspathic porcelain are 92–95%, and the ten-year survival rate is 91%. Similarly, Pjetursson et al. [2] reported a ten-year survival rate of approximately 93% for implants supporting fixed prostheses. However, only a limited proportion of patients were free of biological and technical complications, suggesting that survival does not equate to complication-free success. From a biomechanical standpoint, implants differ from natural teeth in that they are rigidly osseointegrated and lack the periodontal ligament (PDL). The PDL provides a viscoelastic interface that dissipates energy and reduces peak stresses transferred to the surrounding bone [3,4]. As Mapar et al. [5] demonstrated, the presence of the PDL significantly decreases the load transmitted to the bone compared to an ankylosed condition, where the PDL is absent.

The success of implant surgery depends on a combination of biological and biomechanical factors. Biological factors include the quality and quantity of bone, the speed and smoothness of healing, the ability to integrate the implant with bone, and the control of inflammation around the implant. Biomechanical factors include primary stability, micromovements at the implant-bone interface, implant geometry, the magnitude and direction of loading, and parafunctional habits [6,7,8]. A retrospective study by Bardis et al. [9] found that biological complications affected 13.5% of implants, while mechanical and technical complications occurred in 28.7% of cases. For an implant to be considered biomechanically reliable, adequate osseointegration and long-term stability must be ensured. Osseointegration is defined as direct contact between living bone and the implant surface, with no fibrous tissue between them. This process enables bone apposition, maturation, and remodeling around the fixture. During function, loads transmitted from the implant to the surrounding bone act as mechanical stimuli that elicit cellular responses via mechanotransduction. These responses regulate bone remodeling, consistent with Wolff’s law and Frost’s mechanostat model [10,11].

Consequently, inadequate mechanical stimulation may lead to disuse-related bone resorption, while excessive stimulation can cause microdamage and an unfavourable remodeling response [12]. In this context, selecting the right material (metallic, polymeric, or ceramic) is critical. Titanium and titanium alloy implants became the clinical standard following the pioneering observations of Sivaswamy et al. [13] owing to their biocompatibility and mechanical reliability. However, titanium-based systems have limitations. For example, the fixture’s gray color may compromise esthetics in the anterior region, particularly in patients with a thin gingival biotype. Additionally, there is the potential for corrosion and tribocorrosion in the oral environment under pH fluctuations and cyclic loading, as discussed by Spies et al. [14]. According to Sağsöz et al. [15], titanium particles and ions can activate immune mediators and trigger hypersensitivity reactions in certain patients, leading to osteolysis and an increased risk of implant failure. From a biomechanical perspective, titanium’s relatively high stiffness, compared with bone, may promote stress shielding and non-physiological load transfer [16,17].

This phenomenon is often described as reduced mechanical stimulation in apical regions combined with stress concentration in the crestal cortical bone. This can contribute to marginal bone loss and remodeling alterations [18]. The magnitude of stress shielding increases with stiffness mismatch (Young’s modulus) between the implant and bone, becoming particularly critical in low-density bone. Rho et al. [19] and Morgan et al. [20] established key density-elasticity relationships showing that small reductions in bone density markedly decrease stiffness and enlarge the implant-bone modulus gap when rigid materials are used. To mitigate this mismatch, polymeric materials, such as polyether ether ketone (PEEK) and, especially, carbon-fiber-reinforced composites (CFR-PEEK), have been proposed [21].

Francis et al. [22] conducted a review summarizing multiple comparative finite element analysis (FEA) studies. These studies showed that CFR-PEEK can reduce stress peaks at the implant–bone interface compared with titanium, thus improving mechanical compatibility. However, unmodified PEEK is bioinert and hydrophobic (contact angle ~80–90°), with lower wettability than titanium, which limits osseointegration [23]. Nevertheless, the surface properties of PEEK can be enhanced by applying titanium or hydroxyapatite coatings, topographical modifications (e.g., sandblasting or etching), chemical treatments (e.g., sulfonation), or physical methods (e.g., plasma or UV exposure). These methods increase hydrophilicity and promote more favorable cellular responses [24,25,26].

Another limitation of titanium is its gray color, which can affect aesthetic outcomes in the anterior region. Ceramic materials, particularly zirconia, provide a tooth-like appearance and favorable biological performance in this context. Neugebauer et al. [27] reviewed the clinical evidence on ceramic implants and reported encouraging survival outcomes. They also suggested potential improvements in soft-tissue integration and establishment of the peri-implant “biologic width.” However, zirconia has an even higher Young’s modulus than titanium, which may increase stress shielding. Therefore, its mechanical behavior should be carefully assessed, especially in low-density bone [28]. Additionally, zirconia is inherently brittle. First-generation designs have been reported to fracture, including coronal fractures, which could potentially compromise clinical outcomes [29].

To address this limitation, the introduction of yttria-stabilized tetragonal zirconia polycrystal (Y-TZP) represented a major advance, as it provides improved mechanical properties and higher fracture toughness than other ceramics, resulting in greater load-bearing reliability [30,31]. In a clinical systematic review, Padhye et al. [32] reported an overall one-year survival rate of 92% for zirconia implants. Moreover, Lee et al. [33] showed that ceramic surfaces may promote a more favorable biological seal, with a connective-tissue component of the biological width more comparable to that of natural teeth, suggesting a potential benefit for osseointegration and soft-tissue stability.

For assessing stress shielding, FEA is particularly valuable because it allows controlled evaluation of variables that are difficult to isolate in vivo, such as material properties, bone density, geometry, and load direction and magnitude, and enables mapping of stress, strain, and strain energy density in both cortical and trabecular bone. Korabi et al. [34] demonstrated that FEA enables direct comparison of implants with different stiffness levels and supports interpretation of how mechanical stimuli are transmitted to bone relative to remodeling-based criteria. Evidence based on stress shielding is broader in orthopedics than in dental implantology. Studies by Ceddia et al. [35,36,37,38] on femoral stems showed that reducing device stiffness and applying structural optimization strategies can mitigate stress shielding and preserve proximal load transfer. This provides a methodological framework that can be transferred to dental applications.

Based on these studies, the present study aims to use FEA to evaluate stress shielding induced by three one-piece implants made of titanium (Ti-6Al-4V), Y-TZP, and CFR-PEEK in low-density bone. The study also aims to quantify how differences in elastic modulus affect the distribution of mechanical stimuli and the potential risk of peri-implant bone resorption.

## 2. Materials and Methods

**Figure 1:**
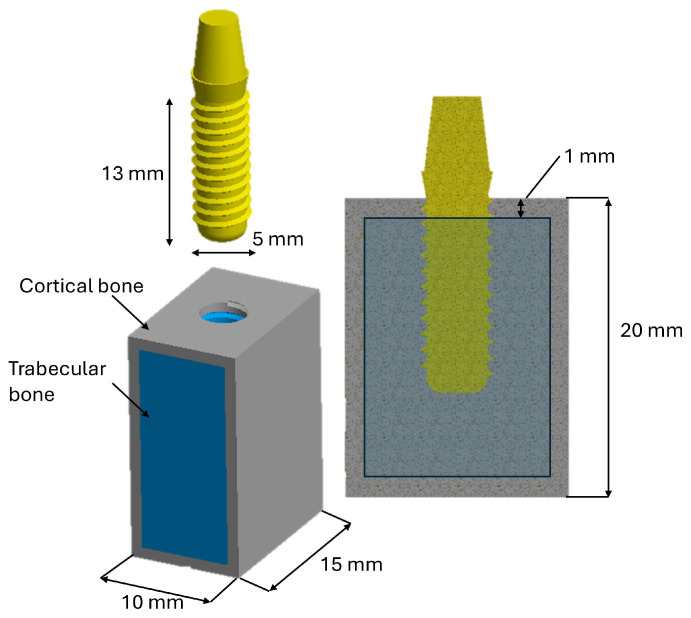
Three-dimensional (3D) representation and geometric dimensions of the implant-to-bone block system.

This study used computer-aided design (CAD) and FEA to virtually model a single-tooth implant system. The three-dimensional (3D) implant geometry was created in Autodesk Inventor 2024 (San Francisco, CA, USA). Figure 1 illustrates the computational model, consisting of a simplified posterior mandibular bone block (molar region) with distinct cortical and trabecular compartments, and a one-piece implant measuring 13 mm in length and 5 mm in diameter. The bone block represents a standardized peri-implant environment used for comparative analyses; its dimensions were selected in line with geometrical ranges commonly used in implant FEA for the molar area [39] and were chosen to be sufficiently larger than the implant to reduce boundary effects while maintaining computational efficiency. All components were exported as .STEP files and subsequently imported into the FEA software ANSYS Workbench 2024 R2 (Workbench, Canonsburg, PA, USA) for the simulation workflow.

### 2.1. Material Properties

All materials, including the bone block and the implant, were modeled as linear-elastic and isotropic, consistent with the common simplifications used in the FEA literature [39,40]. Under isotropy, the mechanical response is fully defined by two independent elastic constants, namely Young’s modulus (E) and Poisson’s ratio (ν), and this approach is widely used to represent both bone and implant components for comparative biomechanical analyses [41,42]. Table 1 summarizes the elastic and mechanical properties of all materials considered in this study.

### 2.2. Boundary and Loading Conditions

**Figure 2:**
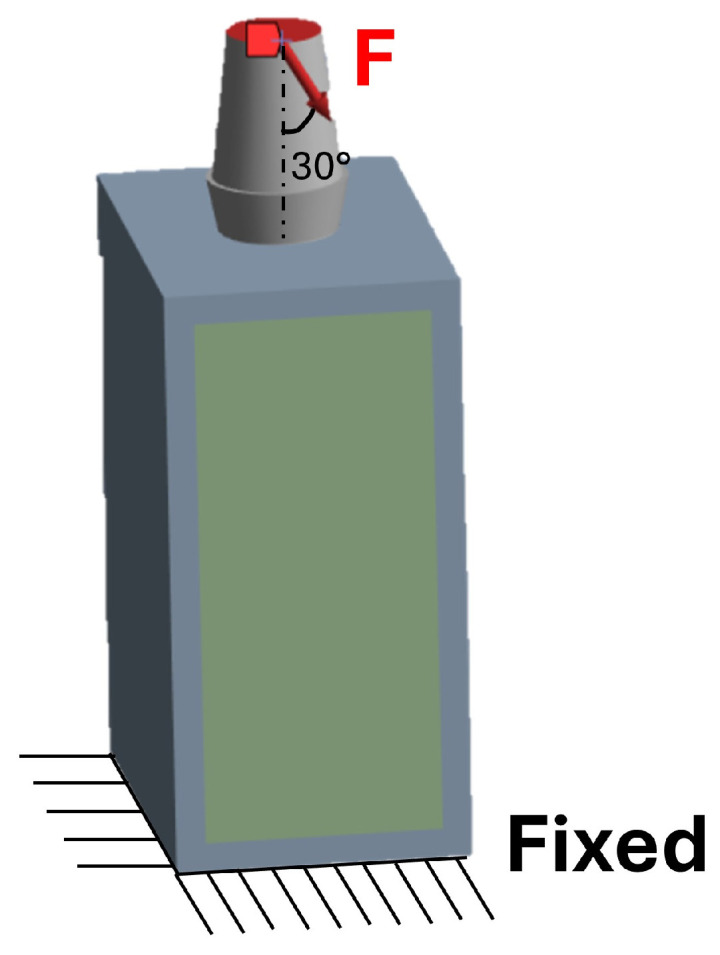
Constraint and load conditions.

For the FEA, files were imported into ANSYS Workbench 2024 R2 (Workbench, Canonsburg, PA, USA). The loading condition consisted of a 200 N static force applied to the top of the implant, inclined at 30° from the buccal to the lingual direction [43]. The inferior surface of the bone block was fully constrained in all degrees of freedom (Figure 2).

For the contact conditions, a bonded interface was defined between the implant and the surrounding bone to simulate complete osseointegration.

### 2.3. Meshing

**Figure 3:**
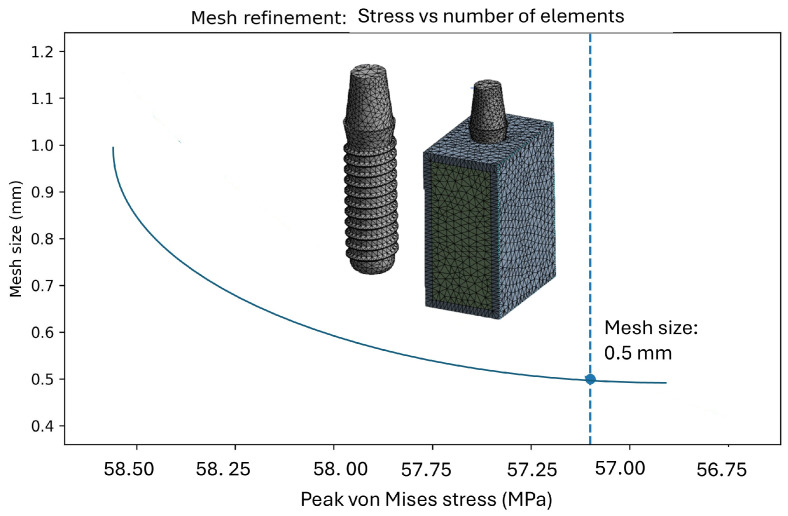
Mesh model with convergence analysis of the implant and bone-implant system.

The mesh was generated using first-order tetrahedral elements. A mesh sensitivity (convergence) assessment was performed by starting with a coarser discretization (characteristic element size of 1.0 mm) and progressively refining the mesh. Based on this analysis, a local element size of 0.5 mm at the implant–bone interface (corresponding to approximately 325,000 elements) was selected, as it yielded stable stress values with negligible changes upon further refinement, indicating convergence. For the remaining bone structures, element sizes in the range of 0.5–1.0 mm were adopted with local refinement at the implant–bone interface. The final mesh comprised approximately 425,000 nodes and ~325,000 elements (Figure 3).

**Figure 4:**
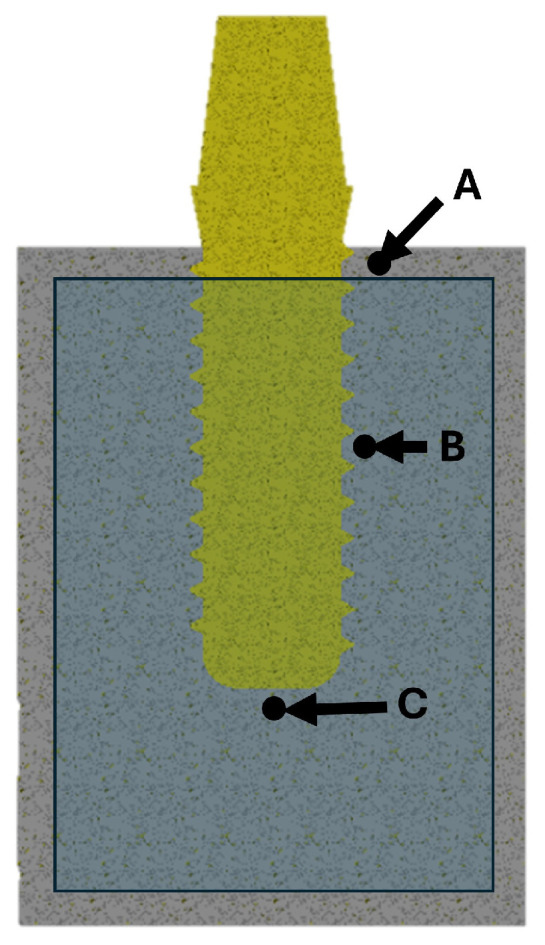
Location of the three points (A, B, and C) showing the crestal section, the middle section, and the apical section, respectively.

To evaluate stress shielding, the study examined the distributions of von Mises stress and strain [44,45,46]. Adequate peri-implant strain levels are essential for sustaining bone metabolic activity and promoting stable remodeling [47,48]. When local strain falls below a minimum threshold, the mechanical stimulus becomes insufficient and may trigger disuse-related remodeling, leading to progressive bone resorption and, in the long term, an increased risk of implant mobility and failure [39,41,42,43,44,45,46,47,48,49,50,51]. According to Kondratiev et al. [51], the strain level associated with a near-equilibrium bone condition is approximately $1000\times 10^{-6}\;\mathrm{mm}/\mathrm{mm}$. Therefore, stress shielding can be quantified using the stress shielding factor (SSF), which measures the reduction in mechanical stimulus in peri-implant bone relative to a reference condition (Equation (1)) [52]:

(1) $$SSF=\frac{\varepsilon_{min}-\varepsilon_{implant}}{\varepsilon_{min}}$$

where $\varepsilon_{\mathrm{implant}}$ denotes the bone strain measured in the peri-implant region. To quantify $\varepsilon_{\mathrm{implant}}$, strain values were extracted from the bone elements located on each transverse section at a constant radial offset of 1 mm from the implant surface. In ANSYS Workbench 2024 R2 (Workbench, Canonsburg, PA, USA), the elements lying along the A–C evaluation path were selected using the Patch Test tool. For each section (A, B, and C), the strain values of the selected elements were averaged to obtain a representative mean peri-implant strain for the crestal, mid-body, and apical regions (Figure 4).

In contrast, $\varepsilon_{min}$ is the minimum strain threshold below which bone is no longer considered to be in a remodeling equilibrium state.

This approach has also been validated in studies by Ceddia et al. [35,36,37,38].

## 3. Results

### 3.1. Von Mises Stress Analysis

FEA was used to evaluate von Mises equivalent stress in cortical and trabecular bone in order to characterize the mechanical response of bone tissue under a 200 N occlusal load. Von Mises stress, derived from elasticity theory, provides a scalar measure that condenses a multiaxial stress state into a single equivalent value. This makes it useful when bone is subjected to complex combinations of compression, tension, and shear.

**Figure 5:**
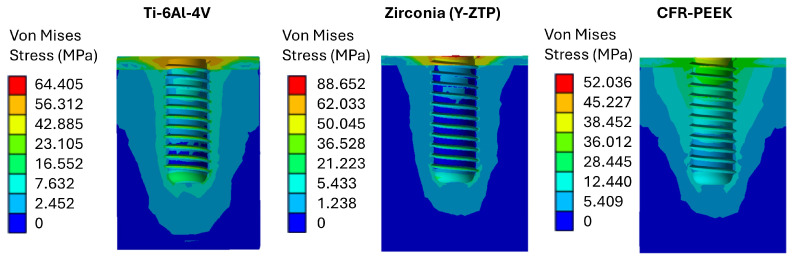
Von Mises stress in bone for the three analyzed implants.

As shown in Figure 5, peak stresses occur in cortical bone.

Because cortical bone is stiffer than trabecular bone, it attracts a larger fraction of the load transferred from the implant. At the same time, it tends to shield the underlying trabecular compartment.

Specifically, the maximum cortical von Mises stress was highest for the Y-TZP implant (87 MPa), followed by the Ti-6Al-4V implant (57 MPa), and the lowest was for the CFR-PEEK implant (37 MPa). This trend aligns with the biomechanics of the system: as the implant’s elastic modulus increases, the stiffness mismatch between the implant and bone increases, thereby promoting greater stress concentration within the crestal cortical region [53].

In contrast, CFR-PEEK promotes more physiologically compatible load transfer because its stiffness is closer to that of bone. This results in lower crestal stress concentration and a more uniform stress distribution along the implant’s longitudinal axis.

Regarding trabecular bone, the highest von Mises stresses were concentrated in the apical region, reaching 17 MPa for Ti-6Al-4V, 6 MPa for Y-TZP, and 16 MPa for CFR-PEEK. This pattern can be explained biomechanically. For highly stiff materials (such as zirconia), a larger portion of the load is borne by the cortical bone and the implant. This reduces the mechanical stimulus transmitted to the trabecular compartment.

Furthermore, when von Mises peak values are compared to threshold values reported in the literature, maximum stresses in the crestal cortical bone are below the commonly assumed compressive strength range for cortical bone (130–230 MPa). For Y-TZP, the maximum stress is 87 MPa; for Ti-6Al-4V, 57 MPa; and for CFR-PEEK, ~37 MPa. These values are shown by Has et al. [54]. This suggests that cortical bone is not expected to reach typical failure levels under static loading. The maximum stresses in the trabecular bone were 17 MPa for Ti-6Al-4V, 6 MPa for Y-TZP, and 16 MPa for CFR-PEEK. Values in the 16–17 MPa range should be interpreted with caution due to the variability of trabecular bone strength, which depends on density and anatomical site. Saini et al. [55] reported a tensile strength range of 22–28 MPa for cancellous/trabecular bone. When this reference is adopted, the predicted stresses remain below the reported strength limits. However, the titanium and CFR-PEEK configurations approach the lower limit of this range, indicating potentially critical, though not necessarily excessive, loading conditions.

### 3.2. Von Mises Strain Analysis

**Figure 6:**
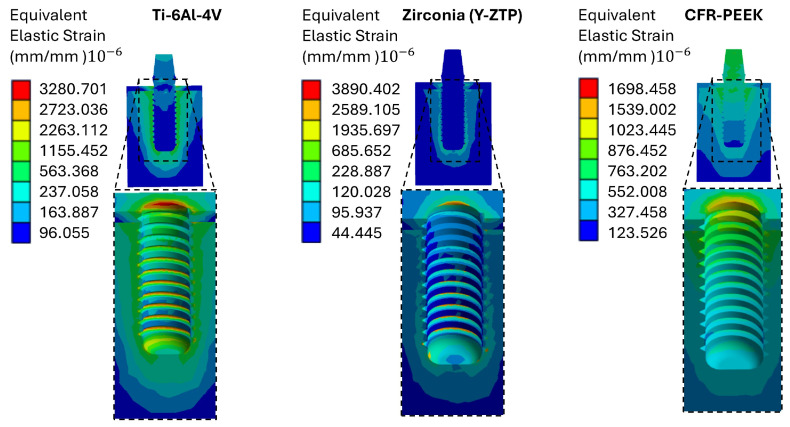
Von Mises strain in bone for the three analyzed implants.

Strain distribution analysis revealed significant differences among the Ti-6Al-4V, Y-TZP, and CFR-PEEK implant systems. Under oblique loading, the peak cortical bone strain was 3103 µε for the titanium system and 3450 µε for the Y-TZP system, whereas the CFR-PEEK system produced lower values (1523 µε). These strain levels can be interpreted according to classic mechanostat thresholds: (i) <100 µε: disuse, (ii) 100–2000 µε: equilibrium (“lazy zone”), (iii) 2000–4000 µε: mild overload that may stimulate bone formation, and (iv) >4000 µε: pathological overload with an increased risk of microdamage and fracture [56]. Based on these results, Ti-6Al-4V and Y-TZP placed cortical bone strain within the mild overload range (with Y-TZP closer to the pathological threshold), while CFR-PEEK yielded strains within the equilibrium zone. This shows a more moderate mechanical stimulus at the cortical level (Figure 6).

### 3.3. Stress Shielding Analysis

To assess stress shielding, bone strain values were extracted from the transverse sections defined by points A–C, as shown in Figure 4. For each section, the mean bone strain was calculated within a region located at 1 mm from the implant surface (Table 2).

**Figure 7:**
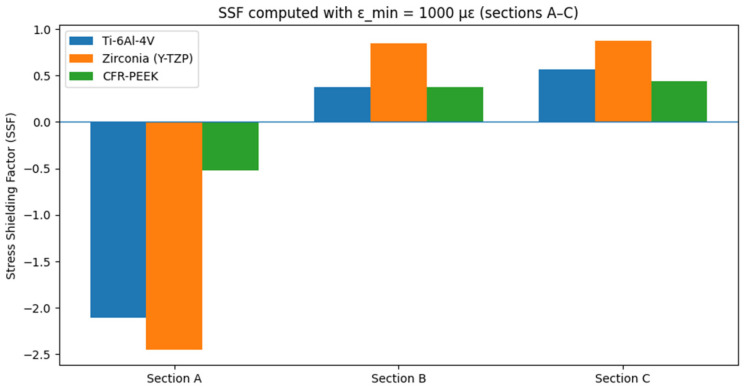
Representation of the stress shielding factor (SSF), calculated in sections A, B, and C, with the three implants made up of different materials.

The SSF was then computed using Equation (1), and the results are shown in Figure 7.

As shown in Figure 7, stress shielding happened mostly in Sections B and C. In both sections, the Y-TZP implant exhibited the highest shielding, with SSF values of 0.844 in Section B and 0.877 in Section C. This shows a significant decrease in the strain transferred to the bone. Ti-6Al-4V showed an intermediate level of shielding (SSF = 0.380 in Section B and SSF = 0.568 in Section C), while CFR-PEEK yielded lower SSF values (SSF = 0.375 in Section B and SSF = 0.437 in Section C). This is consistent with greater load sharing, as its stiffness more closely matches that of bone. In Section A, SSF was negative for all materials because the local strain exceeded 1000 µε. Therefore, no stress shielding was seen in the crestal region.

## 4. Discussion

Rehabilitation of missing teeth with implant-supported prostheses is currently regarded as one of the most established treatment options for edentulous patients [57,58]. However, long-term predictability depends on maintaining an appropriate balance between the biological response and the mechanical environment [59].

Once osseointegration is achieved, peri-implant bone is exposed to complex stress states that are strongly influenced by material properties, implant geometry, and loading direction [60]. Owing to this complexity, FEA has become a widely adopted tool in implant dentistry to numerically investigate the factors governing stress and strain distributions within bone tissue [61,62,63].

In the present study, von Mises equivalent stress and strain, together with the SSF, demonstrated a clear material-dependent response. Cortical bone emerged as the most highly loaded region, with peak stresses of 87 MPa for Y-TZP, 57 MPa for Ti-6Al-4V, and ~37 MPa for CFR-PEEK. These findings indicate a more pronounced crestal stress concentration in the stiffer materials and more uniform load transfer along the implant axis in CFR-PEEK. Consistently, peak cortical strains were 3450 µε (Y-TZP), 3103 µε (Ti-6Al-4V), and 1523 µε (CFR-PEEK). This trend can be explained biomechanically: implants with higher elastic modulus tend to attract a greater portion of the load and stiffen the crestal region, thereby amplifying strain gradients in cortical bone while reducing strain transfer to deeper peri-implant regions (i.e., the apical zone).

Similarly, the study by Lee et al. [64] investigated how the stiffness mismatch of implant materials contributes to stress shielding, showing that reinforced polymer-based materials can reduce the implant–bone modulus gap and, consequently, mitigate the loss of mechanical stimulus in specific peri-implant regions. Sarot et al. [65] compared titanium and CFR-PEEK implants (30% carbon fibers), reporting differences in stress distribution within the supporting bone and suggesting that CFR-PEEK may reduce interfacial stress peaks due to its more compliant elastic response. Schwitalla et al. [66] further demonstrated that unreinforced PEEK can exhibit higher stress peaks and larger deformations than reinforced solutions, whereas continuous-fiber systems (e.g., Endolign^®^) show stress patterns more comparable to titanium, highlighting that fiber architecture and reinforcement fraction are key determinants of mechanical behavior.

These findings provide a consistent framework for interpreting the present stress shielding results across materials. Specifically, Y-TZP, being the stiffer material, reduces deformation in Sections B–C (higher SSF), whereas CFR-PEEK attenuates strain to a lesser extent (lower SSF), consistent with a more gradual load transfer along the implant body. Consistently with this interpretation, Haseeb et al. [67] reported that under both vertical and 30° oblique loading, CFR-PEEK effectively redistributes mechanical stress from the crestal region toward the apical region, thereby improving load sharing along the implant length. Moreover, Mondragon et al. [68], comparing CFR-PEEK and titanium implants across different prosthetic configurations, reported that CFR-PEEK mitigates the adverse effects of the lateral component of occlusal loading, thereby reducing crestal stress peaks.

From a clinical standpoint, however, material selection cannot be driven solely by biomechanical advantages, because osseointegration, primary and secondary stability, soft-tissue response, and the risk of technical complications are also critical determinants of treatment success. Although clinical studies and systematic reviews report high short- to mid-term survival rates for zirconia, these rates vary, due to differences in implant design and surface characteristics [69,70,71,72]. Hashim et al. [73] reported an overall survival rate of approximately 92% for one-piece zirconia implants. Further supporting the promising performance of ceramic implants are Kohal et al. [74] and prospective clinical investigations by Balmer et al. [75], though they indicate that the evidence base remains less mature than that for titanium, particularly with respect to long-term follow-up.

More specifically, Spies et al. [76] reported a one-year survival rate of 88.9% for alumina-toughened zirconia (ATZ) implants, with a mean marginal bone loss of 0.77 mm, highlighting that zirconia can be clinically viable but may remain sensitive to mechanical and design-related factors. In addition, Gahlert et al. [77] conducted failure analyses of fractured zirconia implants, emphasizing the intrinsic brittleness of ceramics and the importance of minimizing stress concentrations—an aspect consistent with the higher crestal loading observed for zirconia in the present study.

Within this framework, CFR-PEEK appears biomechanically attractive because it reduces stiffness between the implant and the bone [78]. This limits stress shielding in deeper peri-implant regions. However, the literature consistently indicates that PEEK’s low osteoconductivity and reduced wettability relative to titanium are major limitations that may impair osseointegration and secondary stability unless the material is appropriately engineered.

### Limitations

The present findings should be interpreted in light of the main simplifications of the adopted FEA framework. The mandible was represented as a standardized posterior bone block with predefined cortical thickness and linearly elastic properties, which does not capture patient-specific anatomy or spatial variability in bone quality.

The implant–bone interface was idealized as fully bonded, whereas partial contact and micromotions may occur clinically and can modify load transfer. In addition, loading was simplified to a static inclined force, without explicitly modeling the crown and occlusal contacts, whereas real mastication is multidirectional and cyclic.

Finally, numerical results depend on modeling choices (mesh, boundary conditions, model size, and simplified material descriptions). Moreover, SSF magnitude and the shielding/no-shielding boundary are inherently dependent on the selected mechanostat threshold ε_min_, particularly in regions where strains are close to the threshold.

Overall, these assumptions may affect absolute stress/strain values, but are not expected to alter the comparative trends among implant configurations. Future studies will specifically aim to quantify how each of these modeling choices (bone constitutive behavior, interfacial conditions, physiological/cyclic loading, mesh/boundary effects, and $\varepsilon_{min}$ selection) affects stress-shielding metrics and will integrate experimental and/or clinical validation to strengthen the quantitative interpretation of stress shielding.

## 5. Conclusions

Finite element simulations showed that, in low-density bone under oblique loading, peri-implant biomechanical behavior is strongly influenced by the implant material. In general, stiffer materials, such as Ti-6Al-4V and Y-TZP, tend to concentrate stresses at the crestal region and reduce mechanical stimulation in deeper bone volumes, thereby increasing the likelihood of stress shielding. Conversely, materials with a stiffness closer to that of bone, such as CFR-PEEK, promote a more homogeneous load transfer and greater load sharing within peri-implant tissues. Clinically, these findings suggest that material selection may help modulate the risk of unfavorable remodeling: in patients with poor bone quality, implants with a closer stiffness match to bone could attenuate crestal stress peaks and limit shielding in deeper regions, potentially supporting better long-term preservation of peri-implant bone volume.

However, clinical decision-making cannot rely on biomechanics alone. Long-term reliability and biological performance remain essential determinants of treatment predictability. Therefore, while these results support the use of FEA as a comparative tool to inform material selection and implant design, they also highlight the need for further experimental and clinical validation under more realistic conditions.

## Figures and Tables

**Table 1:** Mechanical properties of materials.

Material	Young’s Modulus E (GPa)	Poisson’s Ratio ν
Cortical bone	14.8	0.3
Trabecular bone	1.6	0.3
Ti-6Al-4V	110	0.34
Y-ZTP	210	0.31
CFR-PEEK	18	0.39

**Table 2:** Results of the strain calculated in the sections identified by control points A, B, and C.

Sections	Ti-6Al-4V	Y-ZTP	CFR-PEEK
A	3103	3450	1523
B	620	156	625
C	432	123	563

## Data Availability

The original contributions presented in this study are included in the article. Further inquiries can be directed to the corresponding author.

## References

[1] Van den Breemer C.R.G., Cune M.S., Özcan M., Naves L.Z., Kerdijk W., Gresnigt M.M.M. (2019). Randomized clinical trial on the survival of lithium disilicate posterior partial restorations bonded using immediate or delayed dentin sealing after 3 years of function. J. Dent..

[2] Pjetursson B.E., Thoma D., Jung R., Zwahlen M., Zembic A. (2012). A systematic review of the survival and complication rates of implant-supported fixed dental prostheses (FDP s) after a mean observation period of at least 5 years. Clin. Oral Implant. Res..

[3] Hjalmarsson L., Gheisarifar M., Jemt T. (2016). A systematic review of survival of single implants as presented in longitudinal studies with a follow-up of at least 10 years. Eur. J. Oral Implantol..

[4] Schulze F., Lang A., Schoon J., Wassilew G.I., Reichert J. (2023). Scaffold guided bone regeneration for the treatment of large segmental defects in long bones. Biomedicines.

[5] Mapar A., Taheri-Nassaj N., Shen J., Komari O., Sheets C.G., Earthman J.C. (2022). Finite element study of periodontal ligament properties for a maxillary central incisor and a mandibular second molar under percussion conditions. J. Med. Biol. Eng..

[6] Mathur M., Phogat P., Jewariya M., Wan M. (2025). Surface characteristics and their influence on osseointegration: A scientometric analysis with a focus on dental implants. J. Maxillofac. Oral Surg..

[7] Heitz-Mayfield L.J., Salvi G.E. (2018). Peri-implant mucositis. J. Clin. Periodontol..

[8] Jung J., Ryu J.I., Shim G.J., Kwon Y.D. (2023). Effect of agents affecting bone homeostasis on short-and long-term implant failure. Clin. Oral Implant. Res..

[9] Bardis D., Agop-Forna D., Pelekanos S., Chele N., Dascălu C., Török R., Török B., Cristea I., Bardi P.M., Forna N. (2023). Assessment of Various Risk Factors for Biological and Mechanical/Technical Complications in Fixed Implant Prosthetic Therapy: A Retrospective Study. Diagnostics.

[10] Weinkamer R., Hartmann M.A., Brechet Y., Fratzl P. (2004). Stochastic lattice model for bone remodeling and aging. Phys. Rev. Lett..

[11] Martino F., Perestrelo A.R., Vinarský V., Pagliari S., Forte G. (2018). Cellular mechanotransduction: From tension to function. Front. Physiol..

[12] Wei X., Cooper D.M. (2023). The various meanings and uses of bone “remodeling” in biological anthropology: A review. Am. J. Biol. Anthropol..

[13] Sivaswamy V., Vasudevan S. (2022). Dental Implants: An Overview. Dental Implants and Oral Microbiome Dysbiosis: An Interdisciplinary Perspective.

[14] Spies B.C., Sperlich M., Fleiner J., Stampf S., Kohal R.J. (2016). Alumina reinforced zirconia implants: 1-year results from a prospective cohort investigation. Clin. Oral Implant. Res..

[15] Polat Sağsöz N., Murat F., Sevinç Gül S.N., Şensoy A.T., Kaymaz I. (2025). CF-PEEK vs. Titanium Dental Implants: Stress Distribution and Fatigue Performance in Variable Bone Qualities. Biomimetics.

[16] Swalsky A., Noumbissi S.S., Wiedemann T.G. (2024). The systemic and local interactions related to titanium implant corrosion and hypersensitivity reactions: A narrative review of the literature. Int. J. Implant Dent..

[17] Khaohoen A., Sornsuwan T., Chaijareenont P., Poovarodom P., Rungsiyakull C., Rungsiyakull P. (2023). Biomaterials and clinical application of dental implants in relation to bone density—A narrative review. J. Clin. Med..

[18] Gao B., Sun Z., Tong Y., Yu H., Wang F. (2025). Research Progress on Personalized Bone Implants Based on Additive Manufacturing. Micromachines.

[19] Rho J.Y., Hobatho M.C., Ashman R.B. (1995). Relations of mechanical properties to density and CT numbers in human bone. Med. Eng. Phys..

[20] Morgan E.F., Bayraktar H.H., Keaveny T.M. (2003). Trabecular bone modulus–density relationships depend on anatomic site. J. Biomech..

[21] Schwitalla A., Müller W.D. (2013). PEEK dental implants: A review of the literature. J. Oral Implantol..

[22] Francis J.N., Banerjee I., Chugh A., Singh J. (2022). Additive manufacturing of polyetheretherketone and its composites: A review. Polym. Compos..

[23] Najeeb S., Zafar M.S., Khurshid Z., Siddiqui F. (2016). Applications of polyetheretherketone (PEEK) in oral implantology and prosthodontics. J. Prosthodont. Res..

[24] Rupp F., Gittens R.A., Scheideler L., Marmur A., Boyan B.D., Schwartz Z., Geis-Gerstorfer J. (2014). A review on the wettability of dental implant surfaces I: Theoretical and experimental aspects. Acta Biomater..

[25] Gittens R.A., Scheideler L., Rupp F., Hyzy S.L., Geis-Gerstorfer J., Schwartz Z., Boyan B.D. (2014). A review on the wettability of dental implant surfaces II: Biological and clinical aspects. Acta Biomater..

[26] Alqurashi H., Khurshid Z., Syed A.U.Y., Habib S.R., Rokaya D., Zafar M.S. (2021). Polyetherketoneketone (PEKK): An emerging biomaterial for oral implants and dental prostheses. J. Adv. Res..

[27] Neugebauer J., Schoenbaum T.R., Pi-Anfruns J., Yang M., Lander B., Blatz M.B., Fiorellini J.P. (2023). Ceramic Dental Implants: A Systematic Review and Meta-analysis. Int. J. Oral Maxillofac. Implant..

[28] Apratim A., Eachempati P., Salian K.K.K., Singh V., Chhabra S., Shah S. (2015). Zirconia in dental implantology: A review. J. Int. Soc. Prev. Community Dent..

[29] Raić K., Manojlović V. (2025). Corrosion Behavior of Dental Metallic Alloys. Metall. Mater. Data.

[30] Kelly J.R., Denry I. (2008). Stabilized zirconia as a structural ceramic: An overview. Dent. Mater..

[31] Zanocco M. (2016). Raman Spectroscopic Analysis of Zirconia Toughened Alumina Ceramic (ZTA) in Presence of Different Metal Stains and ZTA Retrieval Femoral Heads. https://api.semanticscholar.org/CorpusID:59018484.

[32] Padhye N.M., Calciolari E., Zuercher A.N., Tagliaferri S., Donos N. (2023). Survival and success of zirconia compared with titanium implants: A systematic review and meta-analysis. Clin. Oral Investig..

[33] Lee D.J., Ryu J.S., Shimono M., Lee K.W., Lee J.M., Jung H.S. (2019). Differential healing patterns of mucosal seal on zirconia and titanium implant. Front. Physiol..

[34] Korabi R., Shemtov-Yona K., Dorogoy A., Rittel D.J.S.R. (2017). The failure envelope concept applied to the bone-dental implant system. Sci. Rep..

[35] Ceddia M., Solarino G., Cassano G.D., Trentadue B. (2023). Finite Element Study on Stability in the Femoral Neck and Head Connection to Varying Geometric Parameters with the Relates Implications on the Effect of Wear. J. Compos. Sci..

[36] Ceddia M., Trentadue B. (2023). Evaluation of rotational stability and stress shielding of a stem optimized for hip replacements—A finite element study. Prosthesis.

[37] Ceddia M., Solarino G., Giannini G., De Giosa G., Tucci M., Trentadue B. (2024). A Finite Element Analysis Study of Influence of Femoral Stem Material in Stress Shielding in a Model of Uncemented Total Hip Arthroplasty: Ti-6Al-4V versus Carbon Fibre-Reinforced PEEK Composite. J. Compos. Sci..

[38] Ceddia M., Trentadue B. (2024). A review of carbon fiber-reinforced polymer composite used to solve stress shielding in total hip replacement. AIMS Mater. Sci..

[39] Falcinelli C., Valente F., Vasta M., Traini T. (2023). Finite element analysis in implant dentistry: State of the art and future directions. Dent. Mater..

[40] Delbé K., Doumeng M., Denape J., Mérian T., Berthet F., Marsan O., Chabert F. (2025). Contribution of Raman analysis on tribological study of PEEK reinforced with micro or nano SiC particles. Wear.

[41] Fan K., Ruiz-Hervias J., Gurauskis J., Sanchez-Herencia A.J., Baudín C. (2016). Neutron diffraction residual stress analysis of Al2O3/Y-TZP ceramic composites. Boletín Soc. Española Cerámica Vidr..

[42] Premnath K., Sridevi J., Kalavathy N., Nagaranjani P., Sharmila M.R. (2013). Evaluation of stress distribution in bone of different densities using different implant designs: A three-dimensional finite element analysis. J. Indian Prosthodont. Soc..

[43] Sugiura T., Yamamoto K., Horita S., Murakami K., Tsutsumi S., Kirita T. (2017). Effects of implant tilting and the loading direction on the displacement and micromotion of immediately loaded implants: An in vitro experiment and finite element analysis. J. Periodontal Implant Sci..

[44] Poovarodom P., Rungsiyakull C., Suriyawanakul J., Li Q., Sasaki K., Yoda N., Rungsiyakull P. (2024). Multi-objective optimization of custom implant abutment design for enhanced bone remodeling in single-crown implants using 3D finite element analysis. Sci. Rep..

[45] Klarbring A., Torstenfelt B. (2012). Lazy zone bone remodeling theory and its relation to topology optimization. Ann. Solid Struct. Mech..

[46] Mellal A., Wiskott H.W.A., Botsis J., Scherrer S.S., Belser U.C. (2004). Stimulating effect of implant loading on surrounding bone: Comparison of three numerical models and validation by in vivo data. Clin. Oral Implant. Res..

[47] Carter D.R., Van der Meulen M.C.H., Beaupre G.S. (1996). Mechanical factors in bone growth and development. Bone.

[48] Demenko V., Linetskiy I., Yefremov O., Linetska L., Smetankina N., Kondratiev A. (2025). Regenerated Bone Quality as a Determinant of Bone Turnover and Prognosis in Short Plateau Implants: A Finite Element Study. Prosthesis.

[49] Yang Y., Liu Y., Yuan X., Ren M., Chen X., Luo L., Zheng L., Liu Y. (2023). Three-dimensional finite element analysis of stress distribution on short implants with different bone conditions and osseointegration rates. BMC Oral Health.

[50] Yuan X., Liu Y., Yang Y., Ren M., Luo L., Zheng L., Liu Y. (2023). Effect of short implant crown-to-implant ratio on stress distribution in anisotropic bone with different osseointegration rates. BMC Oral Health.

[51] Kondratiev A., Demenko V., Linetskiy I., Weisskircher H.W., Linetska L. (2024). Evaluation of Bone Turnover around Short Finned Implants in Atrophic Posterior Maxilla: A Finite Element Study. Prosthesis.

[52] Avval P.T., Samiezadeh S., Klika V., Bougherara H. (2015). Investigating stress shielding spanned by biomimetic polymer-composite vs. metallic hip stem: A computational study using mechano-biochemical model. J. Mech. Behav. Biomed. Mater..

[53] Azbari Z.S., Rad M.R., Nahvinejad A., Gilakjani H.A., Khorsandi M. Biomechanical analysis of hip replacement stem design: A finite element analysis. Proceedings of the 2022 29th National and 7th International Iranian Conference on Biomedical Engineering (ICBME).

[54] Has L.C., Orbak R. (2025). Effect of Bone Quality, Implant Length, and Loading Timing on Stress Transmission in the Posterior Mandible: A Finite Element Analysis. Bioengineering.

[55] Saini P., Grover V., Sood S., Jain A., Kalra P. (2023). Evaluation and comparison of three-dimensional finite element analysis of stress distribution in immediately placed and loaded conventional and customized three-dimensional printed dental implants. J. Indian Soc. Periodontol..

[56] Reddy K.U.K., Seth A., Vuppuluri A., Verma P.C., Narala S.K.R., Babu P.J., Saravanan P. (2024). Exploring the bio-mechanical behavior of PEEK and CFR-PEEK materials for dental implant applications using finite element analysis. J. Prosthodont. Res..

[57] Srinivasan M., Kamnoedboon P., Angst L., Müller F. (2023). Oral function in completely edentulous patients rehabilitated with implant-supported dental prostheses: A systematic review and meta-analysis. Clin. Oral Implant. Res..

[58] López C.S., Saka C.H., Rada G., Valenzuela D.D. (2016). Impact of fixed implant supported prostheses in edentulous patients: Protocol for a systematic review. BMJ Open.

[59] Li J., Jansen J.A., Walboomers X.F., van den Beucken J.J. (2020). Mechanical aspects of dental implants and osseointegration: A narrative review. J. Mech. Behav. Biomed. Mater..

[60] Gao X., Fraulob M., Haïat G. (2019). Biomechanical behaviours of the bone–implant interface: A review. J. R. Soc. Interface.

[61] Hosseini-Faradonbeh S.A., Katoozian H.R. (2022). Biomechanical evaluations of the long-term stability of dental implant using finite element modeling method: A systematic review. J. Adv. Prosthodont..

[62] DeTolla D.H., Andreana S., Patra A., Buhite R., Comella B. (2000). The role of the finite element model in dental implants. J. Oral Implantol..

[63] Prados-Privado M., Martínez-Martínez C., Gehrke S.A., Prados-Frutos J.C. (2020). Influence of bone definition and finite element parameters in bone and dental implants stress: A literature review. Biology.

[64] Lee W.T., Koak J.Y., Lim Y.J., Kim S.K., Kwon H.B., Kim M.J. (2012). Stress shielding and fatigue limits of poly-ether-ether-ketone dental implants. J. Biomed. Mater. Res. Part B Appl. Biomater..

[65] Sarot J.R., Contar C.M.M., Cruz A.C.C.D., de Souza Magini R. (2010). Evaluation of the stress distribution in CFR-PEEK dental implants by the three-dimensional finite element method. J. Mater. Sci. Mater. Med..

[66] Schwitalla A.D., Abou-Emara M., Spintig T., Lackmann J., Müller W.D. (2015). Finite element analysis of the biomechanical effects of PEEK dental implants on the peri-implant bone. J. Biomech..

[67] Haseeb S.A., Vinaya K.C., Vijaykumar N., Kumar A.S., Sruthi M.K. (2022). Finite element evaluation to compare stress pattern in bone surrounding implant with carbon fiber-reinforced poly-ether-ether-ketone and commercially pure titanium implants. Natl. J. Maxillofac. Surg..

[68] Martinez-Mondragon M., Urriolagoitia-Sosa G., Romero-Ángeles B., Pérez-Partida J.C., Cruz-Olivares I.M., Urriolagoitia-Calderón G. (2023). Bilinear numerical analysis of the structural behavior of a dental implant applied as a biomaterial carbon fiber reinforced polyether-ether-ketone (CFR-PEEK): A finite element analysis. Dent. Hypotheses.

[69] Roehling S., Schlegel K.A., Woelfler H., Gahlert M. (2018). Performance and outcome of zirconia dental implants in clinical studies: A meta-analysis. Clin. Oral Implant. Res..

[70] Mohseni P., Soufi A., Chrcanovic B.R. (2023). Clinical outcomes of zirconia implants: A systematic review and meta-analysis. Clin. Oral Investig..

[71] Afrashtehfar K.I., Del Fabbro M. (2020). Clinical performance of zirconia implants: A meta-review. J. Prosthet. Dent..

[72] Andreiotelli M., Wenz H.J., Kohal R.J. (2009). Are ceramic implants a viable alternative to titanium implants? A systematic literature review. Clin. Oral Implant. Res..

[73] Hashim D., Cionca N., Courvoisier D.S., Mombelli A. (2016). A systematic review of the clinical survival of zirconia implants. Clin. Oral Investig..

[74] Kohal R.J., Knauf M., Larsson B., Sahlin H., Butz F. (2012). One-piece zirconia oral implants: One-year results from a prospective cohort study. 1. Single tooth replacement. J. Clin. Periodontol..

[75] Balmer M., Spies B.C., Vach K., Kohal R.J., Hämmerle C.H., Jung R.E. (2018). Three-year analysis of zirconia implants used for single-tooth replacement and three-unit fixed dental prostheses: A prospective multicenter study. Clin. Oral Implant. Res..

[76] Spies B.C., Balmer M., Patzelt S.B.M., Vach K., Kohal R.J. (2015). Clinical and patient-reported outcomes of a zirconia oral implant: Three-year results of a prospective cohort investigation. J. Dent. Res..

[77] Gahlert M., Burtscher D., Grunert I., Kniha H., Steinhauser E. (2012). Failure analysis of fractured dental zirconia implants. Clin. Oral Implant. Res..

[78] Rahmitasari F., Ishida Y., Kurahashi K., Matsuda T., Watanabe M., Ichikawa T. (2017). PEEK with reinforced materials and modifications for dental implant applications. Dent. J..

